# Ghrelin decreases sensitivity to negative feedback and increases prediction-error related caudate activity in humans, a randomized controlled trial

**DOI:** 10.1038/s41386-024-01821-6

**Published:** 2024-02-26

**Authors:** Michal Pietrzak, Adam Yngve, J. Paul Hamilton, Anna Asratian, Emelie Gauffin, Andreas Löfberg, Sarah Gustavson, Emil Persson, Andrea J. Capusan, Lorenzo Leggio, Irene Perini, Gustav Tinghög, Markus Heilig, Rebecca Boehme

**Affiliations:** 1 Center for Social and Affective Neuroscience, Department of Biomedical and Clinical Sciences, Linköping University, Linköping, 58183 Sweden; 2 Department of Psychiatry, Linköping University Hospital, Linköping, 58183 Sweden; 3 Center for Medical Imaging and Visualization, Linköping University, Linköping, 58183 Sweden; 4 Division of Economics, Department of Management and Engineering, Linköping University, Linköping, 58183 Sweden; 5 Section on Clinical Psychoneuroendocrinology and Neuropsychopharmacology, Translational Addiction Medicine Branch, National Institute on Drug Abuse Intramural Research Program and National Institute on Alcohol Abuse and Alcoholism, Division of Intramural Clinical and Biological Research, National Institutes of Health, Baltimore, MD 21224 USA; 6 National Center for Health Care Priority Setting, Department of Health Medicine and Caring Sciences, Linköping University, 58183 Linköping, Sweden; 7 Present Address: Department of Medical and Biological Psychology, University of Bergen, Bergen, 5007 Norway

**Keywords:** Reward, Feeding behaviour, Learning and memory, Risk factors, Predictive markers

## Abstract

The stomach-derived hormone ghrelin plays not only a role in feeding, starvation, and survival, but it has been suggested to also be involved in the stress response, in neuropsychiatric conditions, and in alcohol and drug use disorders. Mechanisms related to reward processing might mediate ghrelin’s broader effects on complex behaviors, as indicated by animal studies and mostly correlative human studies. Here, using a within-subject double-blind placebo-controlled design with intravenous ghrelin infusion in healthy volunteers (*n* = 30), we tested whether ghrelin alters sensitivity to reward and punishment in a reward learning task. Parameters were derived from a computational model of participants’ task behavior. The reversal learning task with monetary rewards was performed during functional brain imaging to investigate ghrelin effects on brain signals related to reward prediction errors. Compared to placebo, ghrelin decreased punishment sensitivity (*t* = −2.448, *p* = 0.021), while reward sensitivity was unaltered (*t* = 0.8, *p* = 0.43). We furthermore found increased prediction-error related activity in the dorsal striatum during ghrelin administration (region of interest analysis: *t-*values ≥ 4.21, *p*-values ≤ 0.044). Our results support a role for ghrelin in reward processing that extends beyond food-related rewards. Reduced sensitivity to negative outcomes and increased processing of prediction errors may be beneficial for food foraging when hungry but could also relate to increased risk taking and impulsivity in the broader context of addictive behaviors.

## Introduction

Being hungry alters our mood and behavior. This is reflected in common expressions like the humorous word-creation of “hangry” (combining hungry with angry). From an evolutionary view-point, it intuitively makes sense that a lack of food will trigger specific behaviors that drive us to prioritize foraging for food, potentially even if these behaviors are dangerous (like a hunt) or have negative consequences (like trying a new type of berry). The stomach-derived hormone ghrelin is secreted when the stomach is empty, and its levels decrease shortly after a meal [1]. Ghrelin administration stimulates appetite and food intake via the growth hormone secretagogue receptor (GHSR) [2]. It has therefore been suggested that ghrelin plays a crucial role in meal initiation and food foraging [3]. However, the effects of ghrelin seem to be more complex than simply regulating food intake (for review, see [4]), as are the behaviors humans needed to be exhibited during foraging for food in a natural setting.

Recent ghrelin studies point to an interaction with reward pathways [3]. One key reward pathway is the dopaminergic projection from the ventral tegmental area (VTA) to the nucleus accumbens (often also referred to as ventral striatum (VS)). In animals, it has been shown that the VTA expresses GHSR [5] and that peripheral and central (LDTg) ghrelin administration increases dopamine release within this pathway [6] (but there are also contradicting findings [7]). A seminal paper also showed in rats that ghrelin targets cells within the VTA to modulate dopaminergic neuron activity. Intra-VTA ghrelin infusion increases feeding, so does peripheral ghrelin infusion and the latter is attenuated by the local administration of a GHSR blocker in the VTA [8]. Central and intra-VTA GHSR blockade also reduces the intake of non-food rewarding substances like alcohol [9].

While a large body of evidence supports the hypothesis that ghrelin affects reward-behaviors in rodents, it remains unclear how changes in systemic ghrelin are exactly translated into altered activity in brain reward pathways. Currently it is suggested that ghrelin might be able to access the brain through different systems, that its transport to the brain depends on its systemic levels and physiological state, and that the ghrelin system might be in part acting independently from circulating ghrelin via intrinsic GHSR activity [10]. How circulating ghrelin accesses the human brain has not been directly investigated, and there are conflicting results about the accessibility of circulating plasma ghrelin into the rodent brain, however it is unclear how this translates to the functioning of the ghrelin system in humans [10]. Ghrelin in humans is highly likely transported through the blood-brain and/or blood-cerebrospinal fluid barriers because of its presence in human CSF, and ghrelin concentrations correspond with changes in the energy balance [11].

Human studies have implicated dysfunctions of the reinforcement learning system in several neuropsychiatric conditions, e.g. alcohol and substance use disorder [12, 13], schizophrenia [14], and depression [15]. The human VS has also been shown to be involved in reinforcement learning processes [16–20]. This has been found for both primary rewards (i.e. something necessary for survival, e.g. food) and secondary rewards (i.e. an outcome with a learned value that facilitates the retrieval of a primary reward) [21]. Brain functional magnetic resonance imaging (fMRI) suggests that VS activity is related to prediction errors, i.e. the difference between an expected and an actually experienced outcome [21]. Based on the prediction-error encoded in the VS, dorsal striatum activation then triggers an action and initiates appropriate behaviors [22]. These differential roles of ventral and dorsal parts of the striatum have been coined “critic” and “actor” [23].

Only a few human studies have manipulated ghrelin levels directly by injecting ghrelin, most studies measured plasma ghrelin levels and applied a correlative approach. Results regarding neural reward signals have been ambiguous, with the majority of studies reporting a positive association with ghrelin levels (for review see [24]), while some report no association (e.g. [25]). These discrepancies might stem from differences in both design (e.g., fasted ghrelin levels vs. ghrelin levels after a meal), analysis (e.g., investigating different phases of the reward response), populations under study (e.g., healthy volunteers vs. people with a clinical diagnosis), the clinical status of the patients under investigation (e.g., people with an active disease vs. people in remission), heterogeneity of patient populations, and last but not least, technical and methodological differences on how peripheral ghrelin was measured and what assay was used [26].

So far, only one study has investigated the effect of ghrelin on reward prediction errors: here, injected ghrelin increased neural signals specific to food reward prediction errors in a network including the VS [27]. Prediction errors are needed to learn from previous experiences and update future expectations and actions. However, one should not always adapt behavior directly after experiencing a single unexpected outcome, i.e., the effect of prediction errors on expectation updates should be weighted by factors pertaining to the environment and the individual’s needs. Energy can be one such need, suggesting that ghrelin signaling might mediate the adjustment of prediction-error influence on learning. Consistent with this hypothesis, behavioral studies in animals suggest that ghrelin can affect reward sensitivity and learning from rewards [28].

To better understand the role of ghrelin for reward processing in humans, we performed a within-subject double-blind randomized controlled experimental medicine study to examine the effect of ghrelin on behavior and neural processing during reward processing and decision-making.

Participants received intravenous (IV) ghrelin and matched placebo in two separate counterbalanced sessions and performed several decision-making tasks during fMRI. Here, we report the analysis of a probabilistic reinforcement learning task [29, 30], during which participants have to learn to associate symbols with monetary reward or punishment and to flexibly adjust their behavior when reward-punishment-contingencies change. This task engages the ventral and dorsal parts of the striatum [23]. We have previously reported results from the other tasks in this same study: participants discounted rewards less during the ghrelin session and that neural activity during anticipation of losses was attenuated [31].

We employed computational modeling of behavior to estimate individual sensitivities to rewarding and punishing feedback, and individual prediction errors throughout the task. We hypothesized that IV ghrelin may alter the impact of reinforcement signals (i.e., rewarding or punishing feedback) on learning and behavior. Therefore, we hypothesized to find a difference in sensitivity to reinforcement between ghrelin and placebo sessions. We further hypothesized that there would be differential prediction-error related neural activity in the striatum between the ghrelin and the placebo session.

## Methods

### Participants

**Fig. 1:**
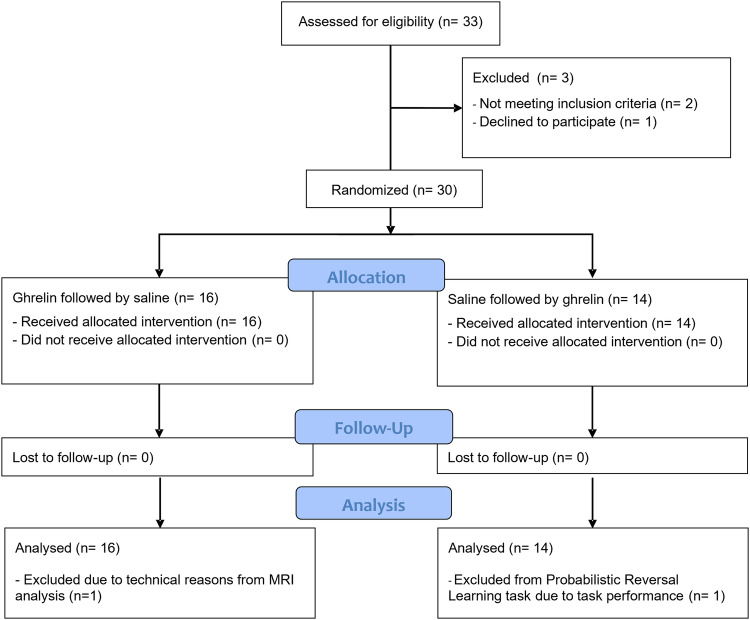
CONSORT diagram of participant flow. Consort Consolidated Standards of Reporting Trials. Participants excluded from analyses were excluded for both sessions.

Thirty healthy volunteers participated in the study (Table 1 and Fig. 1). More details are provided in the previously reported Supplemental Materials [31]. Sample size was determined by a power analysis and stratified by biological sex (50% women, 50% men), given an estimated effect size of Cohen’s *D* ≥ 0.6 at alpha = 0.05. Robust effects of ghrelin on appetite and endocrine measures have repeatedly been detected in smaller sample sizes [2, 32–34]. Inclusion criteria were good health as determined by medical history, physical exam, electrocardiogram (ECG), and clinical assessment of lab tests (see detailed inclusion and exclusion criteria previously reported [31]).

**Table 1:** Sample demographics.

	Descriptive statistics
Female, *n* (%)	15 (50)
Age (years)	26 ± 1.44
Weight (kg)	76 ± 2.03
BMI	23.95 ± 0.51
AUDIT	4.50 ± 0.58
DUDIT	0.13 ± 0.10
NEO-FFI	
Openness	27.33 ± 1.68
Conscientiousness	33.00 ± 1.98
Extraversion	30.13 ± 1.78
Agreeableness	36.57 ± 1.97
Neuroticism	12.13 ± 1.50

Mean ± SEM, *n* = 30.

*BMI* body mass index, *AUDIT* Alcohol Use Disorder Identification Test, *DUDIT* Drug Use Disorder Identification Test, *NEO-FFI* NEO-personality inventory five factor inventory (personality traits were collected for sample description).

### Procedure

This within-subject, cross-over, double-blind, randomized placebo-controlled study was carried out according to GCP, approved by the Swedish Ethics Review Authority (Dnr. 2019-01510) and the Swedish Medicinal Products Agency, and pre-registered as EudraCT 2018-004829-82. Participation consisted of three visits (Fig. S1). Eligibility was assessed at visit 1 using the Mini International Neuropsychiatric Interview [35], the Alcohol Use Disorder Identification Test (AUDIT) [36], and the Drug Use Disorder Identification Test (DUDIT) [37]. Personality traits were assessed with the NEO-PI five factor inventory [33]. Included people signed an informed consent and were randomly assigned to receive ghrelin or placebo at their first or second experimental visit.

Sixteen participants received ghrelin during the first fMRI session and fourteen on the second session. Study staff and participants were blind to the order. Both fMRI visits followed the same procedure prior to scanning. Participants were equipped with two intravenous (IV) catheters, one for infusion and one for collection of blood samples. They received a standardized light lunch (beef patties, red wine sauce, cooked potatoes; energy content: 456.1 kcal; macronutrient content: carbohydrates, 47.6 g; total fat 16.7 g; saturated fat 5.5 g; polyunsaturated fat 1.9 g; monounsaturated Fat 7.8 g; protein 25.4 g; fiber 4.6 g). A baseline blood sample (t1) was collected for analysis of ghrelin levels. Using an MRI simulator (Psychology Software Tools Inc, Pittsburgh, USA), participants trained to keep their head as still as possible and were instructed on the task. A second plasma sample was collected 15 minutes prior to scanning (t2). The infusion was ongoing as participants entered the scanner and remained continuous throughout the whole scan. During a total of 90 min of scanning, anatomical images were collected, and participants performed 12 min of resting state and three decision-making tasks (reversal learning, delay discounting, monetary incentive delay task) in a counterbalanced order. The order was the same during both sessions. A third blood sample was collected at the end of the scan (t3). The results of the other tasks and blood sample analysis have been published separately [31].

### Drug administration

Ghrelin (acyl-ghrelin) was administered continuously as an IV infusion of 5 pmol/kg/min (16.9 ng/kg/min) for up to 4 hours with a mobile MR compatible pump [2, 34, 38–41]. This dose of ghrelin has been used regularly in previous studies [2]. IV ghrelin takes about 60 min to reach steady state and approximately 120 min to reach its full effect. Therefore, the fMRI scanning was carried out between 120–180 min after infusion onset. No treatment-related adverse events occurred. Quality and safety were monitored by an independent individual.

### Reversal learning task

**Fig. 2:**
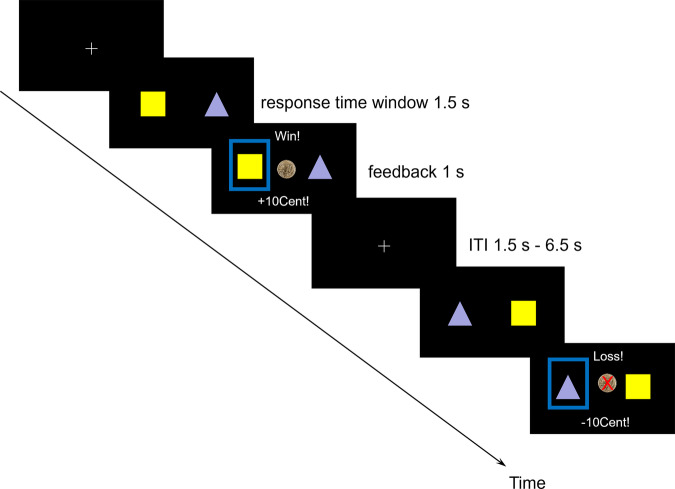
Probabilistic reversal learning task. One trial consists of stimulus presentation with a response time window of 1.5 s, feedback of 1 s, and an inter trial interval (ITI) of 1.5–6.5 s. Choosing the currently ‘good’ stimulus leads with a higher probability (80%) to a reward than the other option (20%). After achieving the criterion of 5 correct answers out of the last 6 trials, the chance of a reversal of the probability distribution is 20% for each following trial until the reversal has occurred or the criterion is no longer fulfilled.

Participants performed a probabilistic reversal learning task ([29, 42], Fig. 2). In this task, the participants are required to monitor reward and punishment contingencies and flexibly adjust their choices to maximize wins. Participants were instructed to try to win as much as possible by choosing the currently best symbol. They were informed that they would receive the amount of money they won in addition to their reimbursement (30 SEK minimum and 300 SEK maximum, at the time of the study 10 SEK ≈ 1€). During fMRI, they performed the task with reversals (200 trials).

The task structure is as follows: Two symbols are presented and can be chosen by button press. One symbol is associated with an 80% chance of monetary reward and a 20% risk of losing money, and vice versa for the other symbol. The probabilities of the two symbols are dependent (i.e. if option *a* has a high probability to provide a reward, option *b* has a high probability to provide a punishment). On each trial, symbols are randomly assigned to the left or right side of the screen. After the participant reaches the learning criterion (5 correct choices out of the last 6 trials), a reversal occurs with a probability of 20% during each trial after the criterion is fulfilled. Each trial consists of stimulus presentation (1.5 s), feedback (1 s) and a jittered, exponentially distributed inter trial interval (1–6.5 s). The symbol has to be chosen within the presentation time. If the time is exceeded, the message “Too slow!” (in Swedish “För långsamt!”) appears. Upon button press, a frame highlights the chosen option, and positive or negative feedback is displayed: a picture of a 10 Swedish Crown coin and the message “You won! +10 Kr” (in Swedish “Du vann! +10 Kr”) or a crossed-out coin and the message “You lost! -10 Kr” (in Swedish “Du förlorade! -10 Kr”).

### fMRI

fMRI data were collected in a 3 Tesla scanner (Prisma, Siemens, Erlangen, Germany). A 64-channel head coil was used to acquire T1-weighted anatomical images (repetition time = 2300 ms; echo time = 2.36 ms; flip angle = 8°; field of view = 288 × 288 mm; acquired/reconstructed voxel resolution = 0.87 × 0.87 × 0.90 mm) and 1030 T2*-weighted echo-planar images (EPI) containing 48 multiband slices (repetition time = 878 ms, echo time = 24 ms, slice thickness 3 mm, field of view 476*476 mm², in-plane voxel resolution 3 mm^2^, flip angle = 56°).

### Data analysis

#### Behavior

Behavioral performance was quantified as the percent of “correct” responses, i.e., choosing the currently better symbol, the number of switches between symbols for consecutive trials, and total number of reversals reached. Participants who achieved less than 5 reversals in a minimum of one of the sessions were excluded from analysis (*n* = 1, f, number of reversals in placebo session: 1, in ghrelin session: 3). Normally distributed measures (percent correct, percent switches, sensitivity to reward, sensitivity to punishment (see below)) were compared using paired t-test, non-normally distributed measures (number of reversals) were compared using Wilcoxon signed rank test. Behavioral data were analyzed using SPSS19 (SPSS Inc., Chicago, IL) and Matlab (The Mathworks, USA).

#### Reinforcement learning model

We used a reinforcement learning model to estimate individual parameters of choice behavior and to calculate prediction errors for fMRI data analysis at a trial-by-trial basis. We were specifically interested in individual sensitivity to reinforcement. The computational model estimates expected outcomes and updates the expected value based on the outcome of the previous trial [43]. Model fitting and selection followed the procedures detailed in [44, 45]. Free parameters (see below) were fitted to best describe the observed behavior using expectation-maximization. We compared eight different model variations to find the one that best captured the actual choice behavior. See supplement for a description of the models and the model selection process. To choose the best-fitting model, we used Bayesian model selection for groups [46] and compared individual log-likelihoods of the models (Table S1). This approach takes the number of parameters into account to avoid overfitting. The model that described the actual choice behavior best in both sessions was a double update model, where the reinforcement sensitivity was estimated separately for win-trials and for loss-trials. Free parameters, which were estimated for each participant and each session individually, were: the learning rate *α*, the reinforcement sensitivity *β* (separately for reward and punishment), and the initial *Q*_*i*_-value (which specifies the first Q-value for one option (a bias to initially choose one over the other stimulus, which increases model fit [47]). These individually estimated free parameters from the best-fitting model were then used to calculate prediction-error values per trial as parametric modulators for the fMRI data analysis.

#### fMRI data

Functional MRI data were analyzed using statistical parametric mapping (SPM12, Wellcome Department of Imaging Neuroscience, London, UK; http://www.fil.ion.ucl.ac.uk/spm) in Matlab R2018b (The MathWorks, Natick, MA, USA). The following preprocessing steps were performed: motion correction, co-registration with the anatomical image, spatial normalization to the MNI-template, and segmentation of the anatomical image using the unified segmentation approach [48]. Normalization parameters were applied to all EPIs, and all images were spatially smoothed with an isotropic Gaussian kernel of 6 mm full width at half maximum. One participant (f) was excluded from MRI analysis due to incomplete data.

For statistical analysis of the blood oxygenation dependent (BOLD) response, SPM’s general linear model was used. To estimate how BOLD responses covaried with prediction errors derived from the learning model, the individually estimated prediction errors were added as parametric modulator to a regressor modeling feedback onset (trial-by-trial, first level model). Motion parameters and a regressor for trials where no answer occurred were included as regressors of no interest. Family-wise-error (FWE) correction at the peak level was used to correct for multiple comparisons for the whole brain, and for small volumes (SVC) based on our a priori regions of interest (ROI). We were specifically interested in the striatum, which has been implicated in reinforcement learning in numerous studies before [21, 49]. We first wanted to see if we can replicate previous results of prediction-error related BOLD signal in the ventral striatum, which were obtained using the same approach of calculating prediction errors with a *Q*-value based reinforcement learning model [20]. Therefore, we used a 6 mm sphere around those previously obtained VS peaks at [14 10 −10] (right) and [−10 12 −10] (left). Paired t-test in SPM was used to compare BOLD responses between the ghrelin and the placebo session. Here, we also included the other striatal subregions caudate and putamen as ROIs, using anatomical masks provided by IBASPM71 [50].

## Results

### Behavior

There was no difference in overall performance between the placebo and the ghrelin session in the reversal task as quantified by basic behavioral measures: the percent of correct choices (placebo: 73 ± 5.1%, ghrelin: 70.9 ± 6.1%, *t* = 1.58, *p* = 0.126), the number of switches (placebo: 56.6 ± 16.2, ghrelin: 58.5 ± 17.9, t = −0.47, *p* = 0.64), or the number of reversals (placebo: 12 ± 3.4, ghrelin: 12 ± 2.9, Z = 0.55, *p* = 0.58). Reaction times did not differ between sessions (placebo: 0.48 ± 0.08, ghrelin: 0.48 ± 0.07, z = −0.78, *p* = 0.44).

### Reinforcement sensitivity

**Fig. 3:**
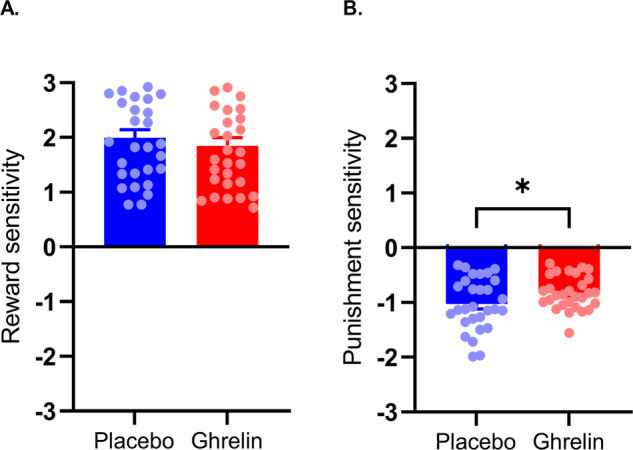
Sensitivity to punishment differs during placebo and ghrelin session. **A** Reward sensitivity did not differ between session [t(28) = 0.8, *p* = 0.43], **B** Punishment sensitivity was reduced during the ghrelin session [t(28) = −2.448, *p* = 0.021].

We compared reinforcement sensitivities between placebo- and ghrelin-session and found that punishment sensitivity was decreased during ghrelin (placebo mean = −1.0288 ± 0.0927, ghrelin mean = −0.8226 ± 0.0592; *t* = −2.448, *p* = 0.021, Cohen’s *d* = −0.455, Fig. 3). Sensitivity to rewards did not differ (placebo mean=1.9943 ± 0.1608, ghrelin mean = 1.8485 ± 0.1587; *t* = 0.8, *p* = 0.43, Cohen’s *d* = 0.148). Sensitivity estimates correlated with each other (reward and punishment sensitivity: placebo: *r* = −0.385, *p* = 0.039, ghrelin: *r* = −0.627, *p* < 0.001). Over sessions, punishment sensitivities correlated (*r* = 0.395, *p* = 0.034), reward sensitivities did not (*r* = 0.258, *p* = 0.177). There was no interaction with BMI when including BMI as a covariate (*p* = 0.163, ηp2 = 0.071). Based on the difference in model parameters, we added an exploratory analysis of behavior, comparing the change in switch-behavior from placebo- to ghrelin-session between trials following win- and loss-feedback. This indicated larger adjustments in the response to loss-feedback in the ghrelin session compared to win-feedback (see supplement).

### Brain imaging

**Fig. 4:**
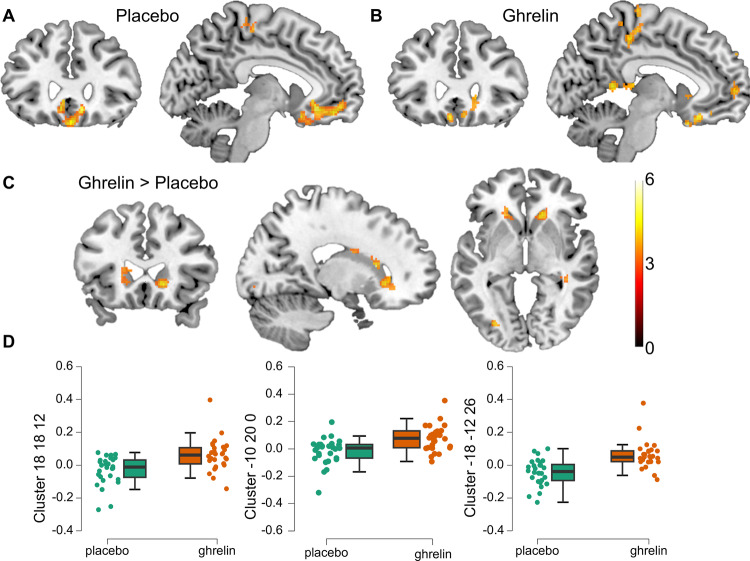
Increased prediction-error related activation during ghrelin infusion. Prediction-error related brain responses during placebo (**A**) and during ghrelin (**B**). **C** Higher prediction-error related response in the bilateral caudate during the ghrelin session than during the placebo session. **D** Average beta parameters from the three caudate clusters containing the significant peaks for visualization purpose. **A** and **B** at [−7 28], **C** at [18 24 −1], all thresholded at *p* < 0.001, clustersize >20 for display purpose.

We found prediction-error related responses during the placebo session in a cluster of the bilateral ventromedial prefrontal cortex ([10 26 −12], *t* = 6.99, *p* = 0.018; [2 50 −12], *t* = 6.95, *p* = 0.02; [2 36 −18], *t* = 6.73, *p* = 0.035; [−8 32 −12], *t* = 6.72, *p* = 0.036; all FWE-corrected for the whole brain, Fig. 4A) and in the ROI of the left VS ([−10 18 −10], t = 3.46, p(SVC) = 0.035).

We then tested for within-subject differences in striatal activation between placebo- and ghrelin-session (Fig. 4). There were no differences at the whole brain level between the sessions. The ROI analysis revealed a significant difference between sessions bilaterally in the dorsal striatum, namely the caudate: prediction errors showed a stronger covariation with the BOLD signal during the ghrelin session compared to the placebo session (left: [−10 20 0], *t* = 4.22, *p* = 0.038; [−18 −12 26], t = 4.14, *p* = 0. 044; right: [18 18 12], *t* = 5.74, *p* = 0.001; [16 24 −4], *t* = 5.12, *p* = 0.005; [20 0 24], *t* = 4.21, *p* = 0.037; Fig. 4C, D displays average beta values for the clusters containing the peaks for visualization only). There were no differences in VS and putamen. There was no interaction with BMI (tested for both the whole brain, and the ROIs) and the inclusion of BMI as covariate did not affect the ghrelin effect in the caudate. There were no significant associations of prediction-error related BOLD signal with individual estimates of punishment sensitivity at the whole brain level or within the ROIs.

## Discussion

We demonstrate that, in a sample of healthy volunteers, ghrelin administration decreases sensitivity to punishment feedback in a reversal learning task and increases prediction-error related BOLD signal in the dorsal striatum.

We have previously reported attenuated striatal responses to loss anticipation in the Monetary Incentive Delay (MID) task, conducted in the same study, during ghrelin administration [31]. However, the MID task is not designed for analysis of specific reinforcement sensitivities. The current finding, using a paradigm specifically designed to model reinforcement learning, shows that punishment sensitivity was lower in the ghrelin session than in the placebo session, and is therefore in line with the prediction of the MID task results.

Our results in humans can be understood along the lines of other preclinical studies where higher ghrelin levels were related to heightened impulsivity [51]. Here, however, we did not find direct indications of increased impulsivity in behavioral measures of reaction times or overall switching between stimuli. Instead, we found a more specific difference in an exploratory analysis suggesting alterations in only the behavior following negative feedback. A previous human study identified an association between fasted ghrelin levels and self-reported reinforcement sensitivities with the same directionality (higher ghrelin levels were associated with more reward sensitivity and less punishment sensitivity, based on questionnaire data) [52]. Becoming less sensitive to negative outcomes fits with the established model of ghrelin as a hormone important to feeding behavior: with higher need for energy, for behavior to be adaptive, the individual should attribute less weight to punishment in order to keep foraging for food. However, this effect might also have the downside of increasing continued use despite negative consequences, which is a hallmark of compulsive substance use or pathological gambling. For both conditions, there is evidence pointing to a potential mediating effect of ghrelin after a ghrelin manipulation obtained by an IV ghrelin administration in people with alcohol use disorder, or an overnight fasting-induced increase in endogenous ghrelin levels, respectively [53, 54].

As described in the introduction, the current state of the field shows conflicting results regarding the access of circulating ghrelin to the brain. Animal studies suggest that ghrelin supports learning mediated by food reward [55], increases dopaminergic activity in mesolimbic reward processing [56], and that GHSRs in the mesolimbic system are necessary for reinforcement learning [57]. Similarly, a recent mouse study found changes in glucose metabolism in the striatum among others areas, however, these alterations were asymmetrical, while our findings are bilateral [58]. It is important to keep in mind that, while most of the ghrelin work in terms of brain sites of action comes from rodent studies, our work here was done in humans. It is still unclear how much the rodent studies translate to humans in terms of central ghrelin sites of action and the ability of the peripheral peptide to reach brain areas related to reward processing.

In humans, the majority of prior studies report a positive association between ghrelin levels and neural responses to reward in striatal and midbrain areas [24]. These studies focused on reward anticipation and consumption, not on reward learning processes. Here, we were specifically interested in prediction-error related responses, which have been found to increase after ghrelin injection in one single study before [27], although only for food-related stimuli (odors). We too find increased reward prediction-error responses during ghrelin administration, but in our case for a monetary reward.

From an evolutionary perspective, it could be hypothesized that ghrelin would not simply increase sensitivity to all rewards and decrease sensitivity to all negative outcomes. This is supported by the recent report of decreased hedonic responses to affective touch during higher amounts of ghrelin [59]. However, monetary rewards are domain-general, as they can be used to gain any type of reward, not just food. A recent meta-analysis demonstrated that primary (e.g. food) and secondary rewards (e.g. money) are processed in overlapping brain networks, with prediction-error processing in the ventral and dorsal striatum for both types of reward stimuli [21]. In our case, monetary reward prediction-error processing during the ghrelin session was associated with heightened dorsal striatum activity. The dorsal part of the striatum is thought to compute appropriate responses following the evaluation of outcomes by the VS [23]. Considering ghrelin’s role in feeding behavior, increased signaling from the ventral to the dorsal part of the striatum could have behavioral consequences that improve foraging, e.g., by increasing behavioral adaptation. This interpretation is also in line with the growing evidence of a role of ghrelin as a survival hormone [60].

We did, however, not find any difference in basic behavioral measures like the number of correct responses or the number of switches between stimuli. This might be due to a ceiling effect, i.e., participants were already performing at a high level during the placebo session and therefore any improvement on flexibility of choice behavior during the ghrelin session did not further improve task performance. The finding of an increased sensitivity to punishment supports this since this parameter is based on the individual choice behavior. It is possible that the model was more sensitive and picked up on behavioral differences, which might be observed in a larger sample or in an experiment with more trials or less clear reinforcement contingencies. Our exploratory comparison of the change in switch behavior between sessions points in this direction, as it suggests that participants changed their response to loss-trials in the ghrelin session more than they did for win-trials. An alternative explanation could be that ghrelin enhances learning from reinforcement signals, but does not necessarily directly affect the behavioral expression, especially for non-food reinforcers [28]. Depending on the specific conditions of a decision-making task, or more generally any given choice situation, varying degrees of flexibility or adaptability of behavior might be beneficial. It remains unclear whether ghrelin increases flexible choice behavior (i.e., exploration versus exploitation) or whether the enhanced caudate activation indicates an increase in effort to identify the most appropriate action.

Ghrelin not only enhances reinforcement learning of food-mediated rewards, but also affects the reinforcing properties of other substances like alcohol, cocaine, and heroin (for reviews, see [61–63]). Emerging work provides translational evidence supporting the same in people with addictive behaviors receiving either an IV ghrelin infusion or a GSHR blocker [54, 64, 65]. In humans, it has been suggested that addictive drugs enhance the computation of prediction errors by increasing activity in the VTA-VS pathway and facilitating the propagation of prediction-error signals from the ventral to the dorsal part of the striatum, a mechanism that could explain the increased acquisition of impulsive choices of addictive substances [66]. Dopaminergic activity in the dorsal striatum in humans has been shown to relate to craving for addictive drugs [67] and to the desire to consume a certain food [68]. This dorsal striatum activity might reflect the readiness or preparation to engage in the actions necessary to obtain the desired stimulus (i.e. food or drug or potentially as in our case a monetary reward) [67]. Our results suggest that ghrelin mediates such an increase in dorsal striatal prediction-error processing.

A potential limitation is that by conducting an intravenous ghrelin infusion, we created a supraphysiological hyperghrelinemia condition which does not necessarily reflect the same physiological conditions as those related to the endogenous ghrelin alone, especially for non-physiological rewards such as those related to alcohol and other addictive drugs. For example, in mice, peripheral sequestration of the endogenous ghrelin peptide blunts weight gain but does not affect cocaine reward, the latter being reduced by GHSR blockade [69]. Similarly, GHSR blockade but not peripheral sequestration of the endogenous ghrelin peptide leads to a reduction in alcohol binge drinking in mice [70]. Together, these findings suggest that the GHSR may play a role in reward processing independently from the endogenous peripheral ghrelin. However, a pharmacological boost of peripheral ghrelin via IV infusion, as we did here, led to an increase in alcohol craving [64] and alcohol self-administration [54] in heavy drinking people with alcohol dependence.

The findings on the role of ghrelin suggest a potential shift in perspective, indicating that this hormone may not primarily influence meal initiation. Instead, it appears to contribute to informing the brain about energy availability, modulating motivation for nutrient search, even influencing the assessment of value [4] and playing a role in mechanisms related to survival [60]. Our findings add to the growing literature on how ghrelin might be a key factor in neurobiological processes that are involved both in behaviors required for survival, i.e., food foraging, and in maladaptive behaviors, including addiction. We showed in humans that ghrelin reduces sensitivity to negative outcomes and enhances dorsal striatal activity in response to reward prediction errors. Future studies should clarify the effect of ghrelin on prediction-error mediated reinforcement signals in the dorsal striatal in the context of non-natural rewards, such as in individuals with substance use disorder.

### Supplementary information

Supplemental Material

## Data Availability

Data cannot be shared as this was not included in the consent form. Unthresholded t- and F-maps for all fMRI analyses can be found at https://identifiers.org/neurovault.collection:16004.
