# Supplementary material for: Ghrelin decreases sensitivity to negative feedback and increases prediction-error related caudate activity in humans, a randomized controlled trial
Source: Neuropsychopharmacology. 2024 Feb 26;49(6):1042–9. doi: 10.1038/s41386-024-01821-6 (PMC11039644; doi:10.1038/s41386-024-01821-6)
Supplement: Supplementary file 1 — Supplemental Material [file 41386_2024_1821_MOESM1_ESM.docx]

**Supplementary Materials**

**Ghrelin decreases sensitivity to negative feedback and increases prediction-error related caudate activity in humans, a randomized controlled trial**

Michal Pietrzak ^1,2,3^, Adam Yngve^1,3^, J. Paul Hamilton ^1,3,7^, Anna Asratian ^1,3^, Emelie Gauffin ^1,2,3^, Andreas Löfberg ^1,2,3^, Sarah Gustavson ^1,2,3^, Emil Persson^4^, Andrea J. Capusan^1,2^, Lorenzo Leggio^5^, Irene Perini ^1,3^, Gustav Tinghög^4,6^, Markus Heilig^1,2,3^, Rebecca Boehme ^1,3^

^1^ Center for Social and Affective Neuroscience, Department of Biomedical and Clinical Sciences, Linköping University, Linköping, 58183 Sweden.

^2^ Department of Psychiatry, Linköping University Hospital, Linköping, 58183 Sweden.

^3^ Center for Medical Imaging and Visualization, Linköping University, 58183 Sweden.

^4^ Division of Economics, Department of Management and Engineering, Linköping University, 58183 Sweden.

^5^ Section on Clinical Psychoneuroendocrinology and Neuropsychopharmacology, Translational Addiction Medicine Branch, National Institute on Drug Abuse Intramural Research Program and National Institute on Alcohol Abuse and Alcoholism, Division of Intramural Clinical and Biological Research, National Institutes of Health, Baltimore, MD 21224, USA.

^6^ National Center for Health Care Priority Setting, Department of Health Medicine and Caring Sciences, Linköping University, 58183 Linköping, Sweden

^7^ Current affiliation: Department of Medical and Biological Psychology, University of Bergen, 5007Norway

**Methods**


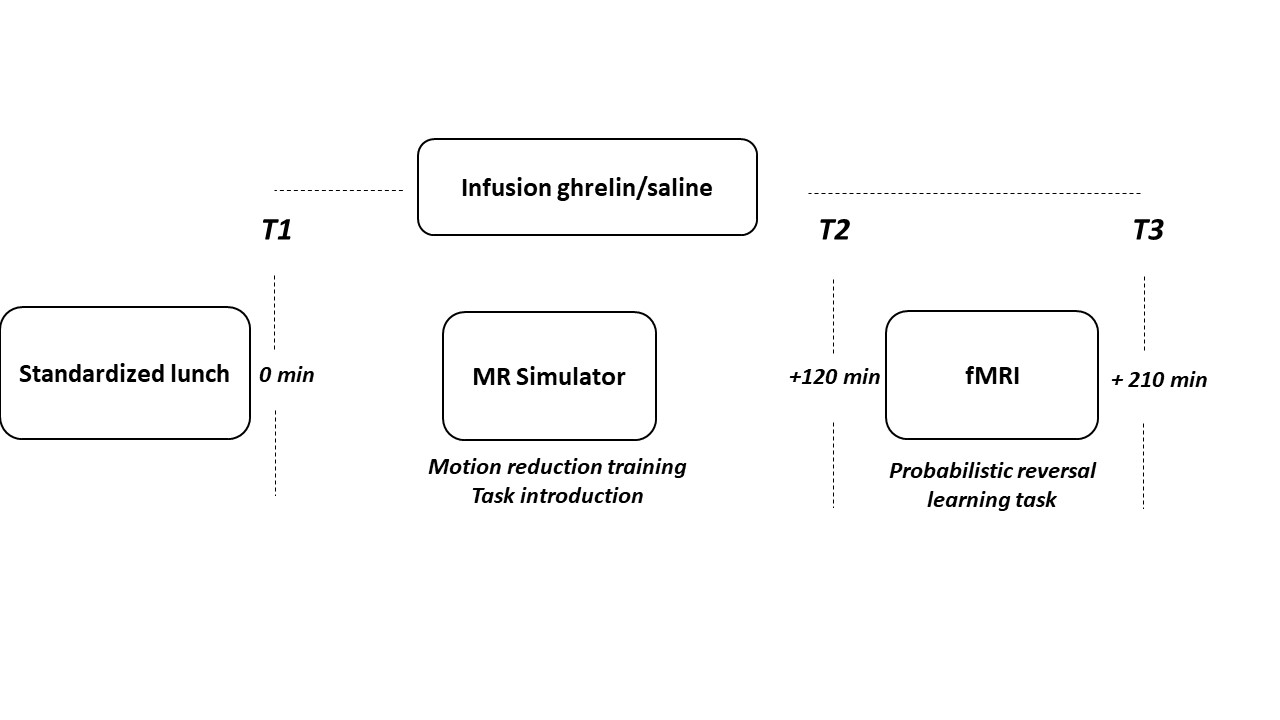


**Figure S1**. Overview of study procedures.

**Model**

At each trial *t*, *Q*-values for the chosen option *c* are adjusted according to the prediction error *δ* from the previous trial *t-1*. The learning rate *α* determines how quickly expectations change with respect to the prediction error *δ.*

(1) $Q_{c,t}=Q_{c,t-1}+\alpha\delta_{Qc,t-1}$

The prediction error is calculated as the difference between the expected outcome value *Q* and the actually received outcome *R*:

(2) $\delta_{Qc,t}=R{}_{c,t}-Q_{c,t}$

A normalized exponential function estimates the probability of choosing one of the symbols over the other based on the *Q*-values of options *a* and *b*. It calculates *p_a_(t),* i.e., the likelihood of choosing option *a* over *b* on trial *t*:

(3) $p_{a}(t)=\frac{\exp(Q_{a}(t))}{\exp(Q_{a}(t))+\exp(Q_{b}(t))}$

We compared different variations of this model. Free parameters, which were estimated for each participant and each session individually, were: the learning rate *α* (equation 1), the reinforcement sensitivity *β* (which replaces R in equation 2)*,* and the initial *Q_i_*-value (which specifies the first Q-value for one option (a bias to initially choose one over the other stimulus, which increases model fit (1))).

We compared eight different model variations to find the one that best captured the actual choice behavior: either one learning rate and one reinforcement sensitivity were estimated for both rewarded and punished trials, or two learning rates and two reinforcement sensitivities were estimated separately. This resulted in four model types: αβ, α2β, 2αβ, 2α2β. We further compared two variations of these models: Single update and double update. While the single update model only updates the expected value of the chosen option *Q_c_*, the double update model also updates the expectation for the unchosen option. We included double-update models based on previous studies with the same structure of anticorrelated stimulus reward probabilities, where these models outperformed single-update models (1-4).

The free parameters were fitted to each individual’s choice behavior by applying expectation-maximization with empirical priors. Model evidence was approximated by integrating out the free parameters over the likelihood by sampling from the prior distribution (5, 6). To choose the best-fitting model, we used Bayesian model selection for groups (7) and compared individual log-likelihoods of the 8 models (Table S1). Model comparison revealed that the double-update-α2β model explained behavior best, i.e., it modelled observed choice behavior of all participants better than chance (based on the likelihood that the observed data are given by the parameters). Individual posterior probability values for this model were above 0.5 for each participant, with a mean of 0.75 ± 0.1 in the placebo condition and 0.72 ± 0.1 in the ghrelin condition, indicating a high likelihood that the model described the behavioral choice data to a high degree.

**Table S1**: Exceedance probabilities (XP) from Bayesian model selection for the eight different models. SU=single update, DU=double update, α=learning rate, β=reinforcement sensitivity

| model | XP (placebo) | XP (ghrelin) |
| --- | --- | --- |
| SU αβ | 0.0017 | 0 |
| SU 2αβ | 0.0007 | 0 |
| SU α2β | 0.0016 | 0 |
| SU 2α2β | 0.0019 | 0.0001 |
| DU αβ | 0.0169 | 0.0053 |
| DU 2αβ | 0. 0717 | 0.0293 |
| DU α2β | **0. 5631** | **0.6498** |
| DU 2α2β | 0.3424 | 0.3155 |

**Exploratory analysis of behavioral session differences**

Since there was a difference in punishment sensitivity as estimated by the model between the sessions, but we did not obtain a significant difference in the typical behavioral parameters, we decided to explore the behavior more closely in order to understand which behavioral differences might underlie the different estimations of reinforcement sensitivity. To do so, we calculated the absolute difference in the number of switches between sessions and compared these for win-trials and for-loss trials.

**Exploratory analysis of sex differences**

Sex differences in prediction error related brain activation were explored in an a posteriori analysis using the SPM function flexible factorial ANOVA with the factors sex (male, female) and session (placebo, ghrelin).

**Results**

**Exploratory analysis of behavioral session differences**

Based on the model-driven assumption of increased punishment sensitivity, we compared the change in the number of switches (i.e. choosing two different symbols in consecutive trials) following win-feedback and loss-feedback. This revealed that the potentially underlying behavioral difference between sessions might be that participants adjusted their responses more to loss-trials in the ghrelin-session (mean change=10.4±9.2) compared to the response following win-feedback (mean change=6.1±7.2; t=-2.6, p=0.015, Fig. S2).

**Figure S2:** Absolute change in switch-behavior between placebo and ghrelin session after win-feedback and after loss-feedback indicates a larger adjustment of behavior after loss-feedback.


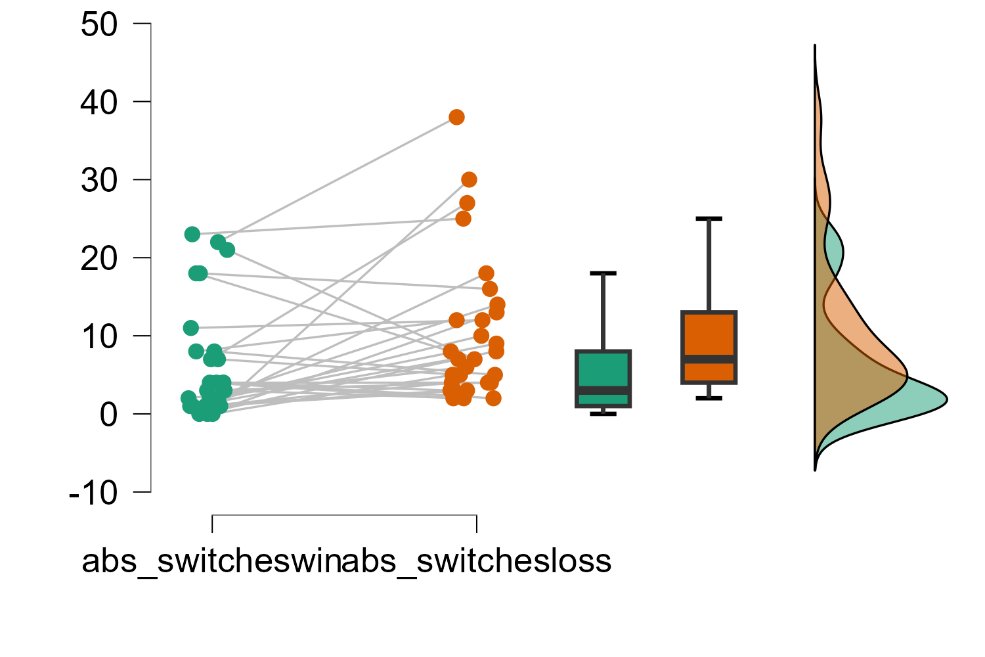


**Exploratory analysis of sex differences and personality traits**

*Behavior*

Conducting a repeated measures ANOVA on punishment sensitivity with sex as a between subject factor showed that, there was a main effect of Intervention (p=0.024, ηp2=0.176), no effect of Sex (p=0.874 ηp2=0.001), and no Intervention x Sex interaction (p=0.501 ηp2=0.017).

Conducting a repeated measures ANOVA on reward sensitivity with sex as a between subject factor showed that, there was no main effect of Intervention (p=0.452, ηp2=0.021), no effect of Sex (p=0.157 ηp2=0.073), and no Intervention x Sex interaction (p=0.955 ηp2=0.00).

Sensitivity analyses were carried out to examine potential contribution from the scores of five domains of personality, neuroticism, extraversion, openness, agreeableness, and conscientiousness, as covariates, but no effect or interactions were found to be significant on punishment sensitivity. We found that a repeated measures ANOVA on reward sensitivity with sex as a between subject factor and Neuroticism as a covariate, showed no main effect of Intervention (p=0.816, ηp2=0.002), no effect of Sex (p=0.329 ηp2=0.040), but a significant effect of Neuroticism (p=0.037, ηp2=0.170), and no Intervention x Sex interaction (p=0.283 ηp2=0.048), and no Intervention x Neuroticism interaction (p=0.733, ηp2=0.005), nor Intervention x Sex x Neuroticism interaction (p=0.936, ηp2=0.000). No significant correlations were found between Neuroticism and reward sensitivity.

*Brain*

There was a main effect of group in (Table S2). This effect was driven by males showing more prediction error related activity than females. However, there was no group by session interaction on the whole brain level or for any of our ROIs.

**Table S2**: Main effect of sex for prediction error related activation. k=clustersize

| Region | Hemisphere | k | x | y | z | F | p (FWE-corr) |
| --- | --- | --- | --- | --- | --- | --- | --- |
| Superior parietal gyrus | R | 16 | 20 | -60 | 70 | 118.75 | 0.000 |
| Supreamarginal gyrus | R | 3 | 42 | -36 | 34 | 66.21 | 0.003 |
| Putamen | R | 5 | 28 | 10 | -2 | 65.74 | 0.003 |
| Cerebellum | R | 6 | 38 | -58 | -24 | 58.15 | 0.010 |
|  | L | 2 | -36 | -62 | -24 | 54.85 | 0.016 |
| Inferior occipital lobe | L | 1 | -38 | -66 | -6 | 52.41 | 0.025 |
| Middle frontal gyrus | R | 1 | 36 | 2 | 64 | 51.76 | 0.028 |
| Superior parietal gyrus | R | 1 | 32 | -60 | 66 | 49.94 | 0.038 |
| Middle occipital gyrus | L | 1 | -38 | -66 | -2 | 49.72 | 0.039 |

**References**

1. F. Schlagenhauf *et al.*, Striatal dysfunction during reversal learning in unmedicated schizophrenia patients. *Neuroimage* **89**, 171-180 (2014).

2. T. U. Hauser *et al.*, Role of the medial prefrontal cortex in impaired decision making in juvenile attention-deficit/hyperactivity disorder. *JAMA psychiatry* **71**, 1165-1173 (2014).

3. J. Glascher, A. N. Hampton, J. P. O'Doherty, Determining a role for ventromedial prefrontal cortex in encoding action-based value signals during reward-related decision making. *Cerebral cortex* **19**, 483-495 (2009).

4. R. Boehme *et al.*, Reversal learning strategy in adolescence is associated with prefrontal cortex activation. *European Journal of Neuroscience* **45**, 129-137 (2017).

5. Q. J. Huys *et al.*, Disentangling the roles of approach, activation and valence in instrumental and pavlovian responding. *PLoS computational biology* **7**, e1002028 (2011).

6. Q. J. Huys *et al.*, Bonsai trees in your head: how the pavlovian system sculpts goal-directed choices by pruning decision trees. *PLoS Comput Biol* **8**, e1002410 (2012).

7. K. E. Stephan, W. D. Penny, J. Daunizeau, R. J. Moran, K. J. Friston, Bayesian model selection for group studies. *NeuroImage* **46**, 1004-1017 (2009).
